# Diphtheritis; a Concise Historical and Critical Essay on the Late Epidemic Pseudo Membraneous Sore-Throat, of California, (1856-7) with a Few Remarks Illustrating the Diagnosis, Pathology and Treatment of the Disease

**Published:** 1860-06

**Authors:** 


Diphtheritis: A concise Historical and Critical Essay on the late Epidemic Pseudo-Membranous Sore Throat, of California, (1856-7) with a few remarks illustrating the Diagnosis, Pathology, and treatment of the Disease. By V. J. Fourgeand, M. D. Sacramento: 1858.

We have recently received a monograph, with the foregoing title, containing 44 pages, well filled with interesting facts and observations, concerning a disease that has attracted much attention during the last few years. We place the Essay on record as an important contribution to the literature of that subject.

